# From Infection to Constriction: Successful Surgical Resolution of Constrictive Pericarditis Following Purulent Pericarditis

**DOI:** 10.7759/cureus.81449

**Published:** 2025-03-30

**Authors:** Vasileios Leivaditis, Sofien Ayed, Ece Özsoy, Henning Lausberg, Athanasios Papatriantafyllou, Francesk Mulita, Nikolaos G Baikoussis, Volker Windmüller, Manfred Dahm

**Affiliations:** 1 Department of Cardiothoracic and Vascular Surgery, Westpfalz-Klinikum, Kaiserslautern, DEU; 2 Department of Cardiology, Westpfalz-Klinikum, Kaiserslautern, DEU; 3 Department of Surgery, General University Hospital of Patras, Patras, GRC; 4 Department of Surgery, General Hospital of Eastern Achaia - Unit of Aigio, Aigio, GRC; 5 Department of Cardiac Surgery, Ippokrateio General Hospital of Athens, Athens, GRC

**Keywords:** cardiac catheterization, constrictive pericarditis, heart failure, pericardiectomy, purulent pericarditis

## Abstract

Constrictive pericarditis (CP) is a rare but serious condition characterized by pericardial fibrosis and impaired ventricular filling, often resulting in progressive heart failure. Infectious pericarditis, particularly purulent forms, is a severe etiology requiring early recognition and intervention. A 64-year-old male with a history of *Staphylococcus aureus* pericarditis presented with worsening dyspnea and signs of right heart failure. Imaging revealed a thickened, fibrotic pericardium with mild effusion, while cardiac catheterization confirmed CP with equalized diastolic pressures and a dip-plateau phenomenon. Given his clinical deterioration, he underwent subtotal pericardiectomy with pericardial reconstruction. Intraoperatively, severe adhesions were noted, necessitating extensive pericardial resection. The patient showed rapid postoperative improvement, with a resolution of heart failure symptoms and normalization of right ventricular function. This case highlights the importance of timely diagnosis and surgical intervention in CP following infectious pericarditis. Pericardiectomy remains the definitive treatment, with early recognition being key to optimizing patient outcomes.

## Introduction

Constrictive pericarditis (CP) is a chronic inflammatory condition characterized by fibrosis, thickening, and in some cases, calcification of the pericardium, leading to impaired diastolic filling of the heart [1]. This pathological process results in restricted ventricular expansion, causing elevated systemic venous pressures, reduced cardiac output, and ultimately, symptoms of right-sided heart failure [2]. While CP can arise from a variety of etiologies, including viral or bacterial infections, prior cardiac surgery, radiation therapy, autoimmune diseases, and idiopathic causes, infectious pericarditis remains a particularly aggressive and challenging subset, often requiring early recognition and intervention [3].

The clinical presentation of CP is often insidious and nonspecific, leading to diagnostic delays. Patients typically exhibit progressive exertional dyspnea, peripheral edema, ascites, and signs of systemic congestion, mimicking other causes of heart failure. Key hemodynamic findings include elevated and equalized diastolic pressures across all cardiac chambers and the classic dip-plateau (square root) sign on right ventricular pressure tracings [2]. Echocardiography, particularly Doppler imaging, is instrumental in assessing ventricular interdependence (the phenomenon where the filling of one ventricle affects the filling of the other due to the noncompliant pericardium), abnormal septal motion, and restrictive filling patterns. Cardiac catheterization remains the gold standard for confirming the diagnosis by providing direct hemodynamic measurements, while computed tomography (CT) and cardiac magnetic resonance imaging (MRI) help assess pericardial thickening, calcification, and secondary myocardial involvement [3].

Despite initial medical therapy aimed at reducing congestion with diuretics, definitive management of CP typically involves pericardiectomy, the surgical removal of the diseased pericardium, to restore normal ventricular filling [1]. Pericardiectomy is associated with significant perioperative risks, particularly in patients with advanced disease and compromised myocardial function [4]. The surgical approach requires meticulous dissection of dense pericardial adhesions while avoiding injury to the underlying myocardium, coronary arteries, and phrenic nerves. In cases of infectious pericarditis, particularly purulent pericarditis, subtotal pericardiectomy and pericardial reconstruction may be necessary to prevent postoperative complications, such as recurrence or excessive scar formation [5].

Here, we present the case of a 64-year-old male patient with a history of purulent pericarditis due to *Staphylococcus aureus *who subsequently developed CP, leading to hemodynamic compromise. The case highlights the diagnostic challenges, the role of multimodal imaging, and the importance of early surgical intervention in preventing irreversible heart failure. Furthermore, we provide a detailed discussion of the intraoperative findings, postoperative management, and the role of pericardial reconstruction in optimizing long-term outcomes for patients with CP.

## Case presentation

A 64-year-old male patient with a history of arterial hypertension, type 2 diabetes mellitus, and previous tobacco use presented with progressive dyspnea, orthopnea, and bilateral lower limb edema. He had a prior history of purulent pericarditis caused by *S. aureus*, which had been managed with emergency pericardiocentesis and targeted antibiotic therapy. Despite initial clinical improvement, he developed recurrent pericardial effusion and signs of cardiac decompensation, prompting further evaluation.

**Figure 1:**
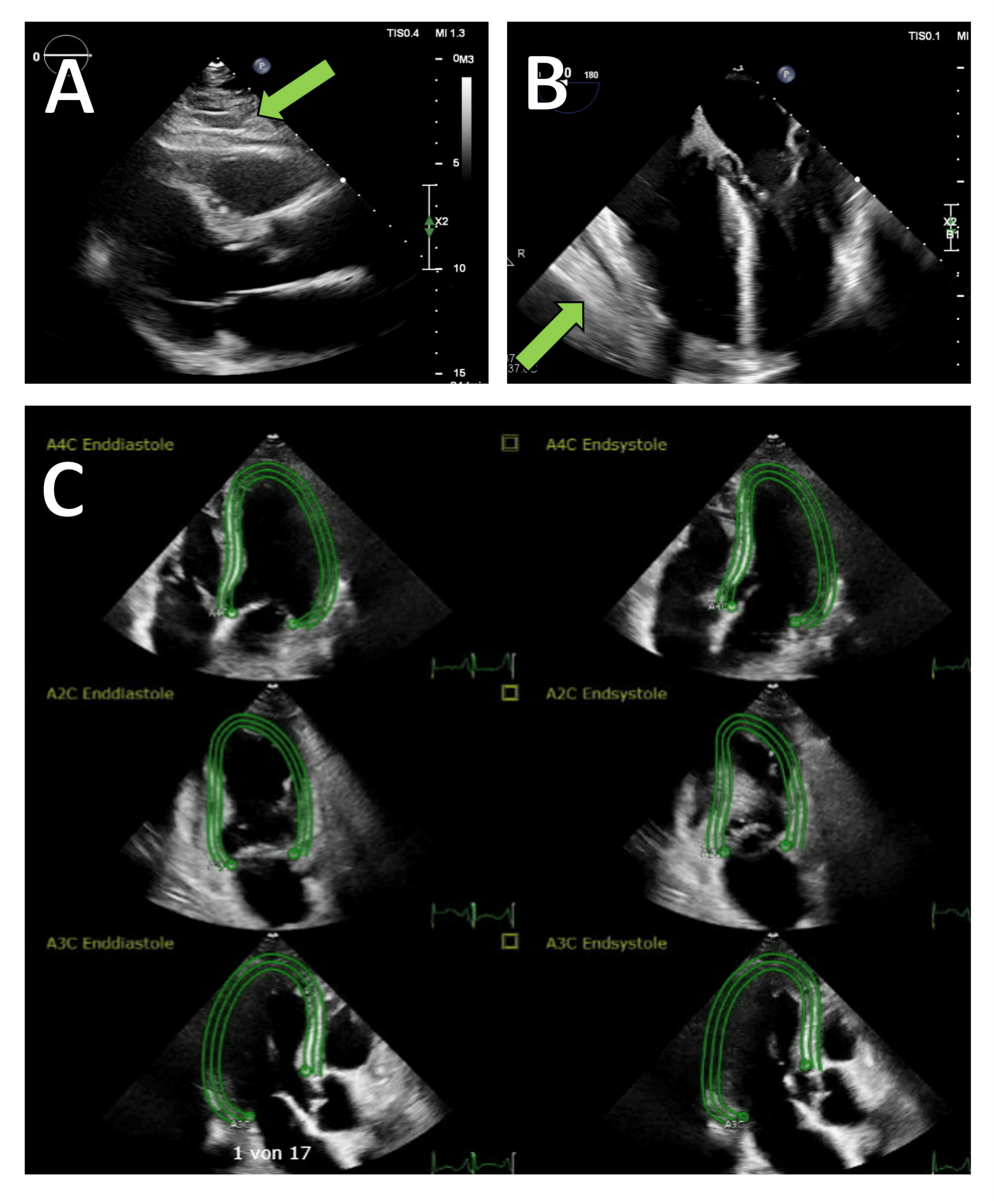
Echocardiographic assessment of constrictive pericarditis. (A) Long-axis transthoracic echocardiographic view showing pericardial thickening (arrow). (B) Transesophageal four-chamber view highlighting the constrictive pericardium (arrow). (C) Doppler evaluation demonstrating a restrictive filling pattern.

**Figure 2:**
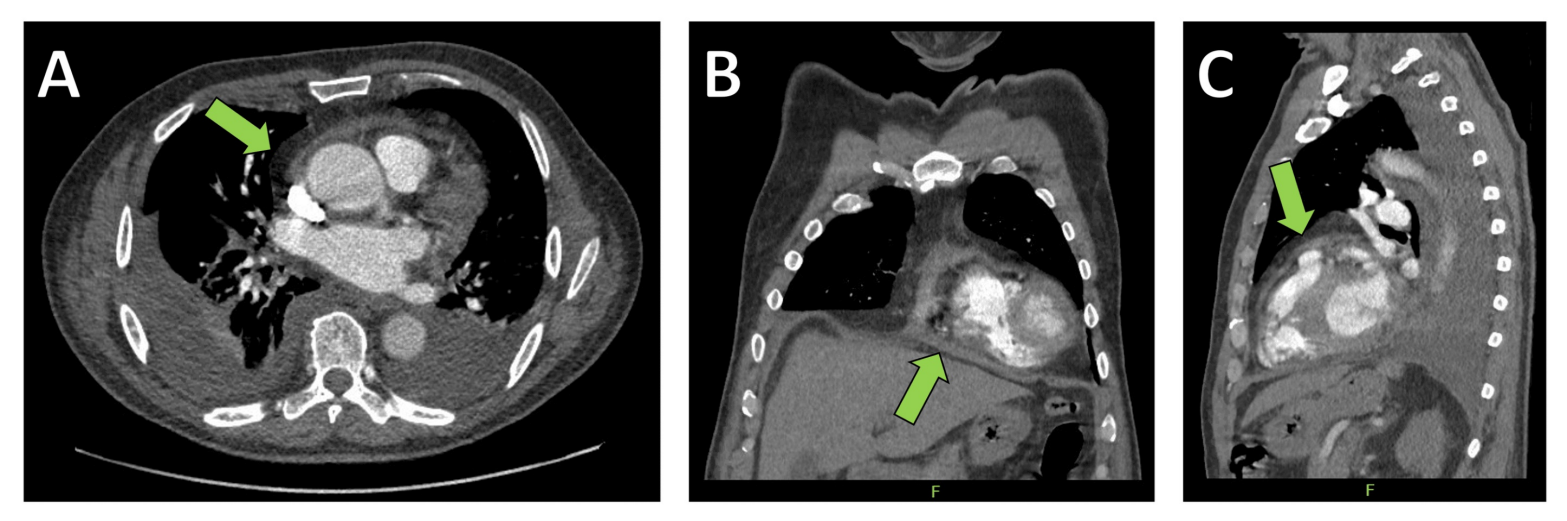
Computed tomography (CT) imaging confirming the diagnosis of constrictive pericarditis. (A) Transverse cross-sectional view demonstrating circumferential pericardial thickening (arrow). (B) Coronary reconstruction highlighting pericardial involvement (arrow). (C) Sagittal reconstruction illustrating pericardial calcifications and associated findings (arrow).

**Figure 3:**
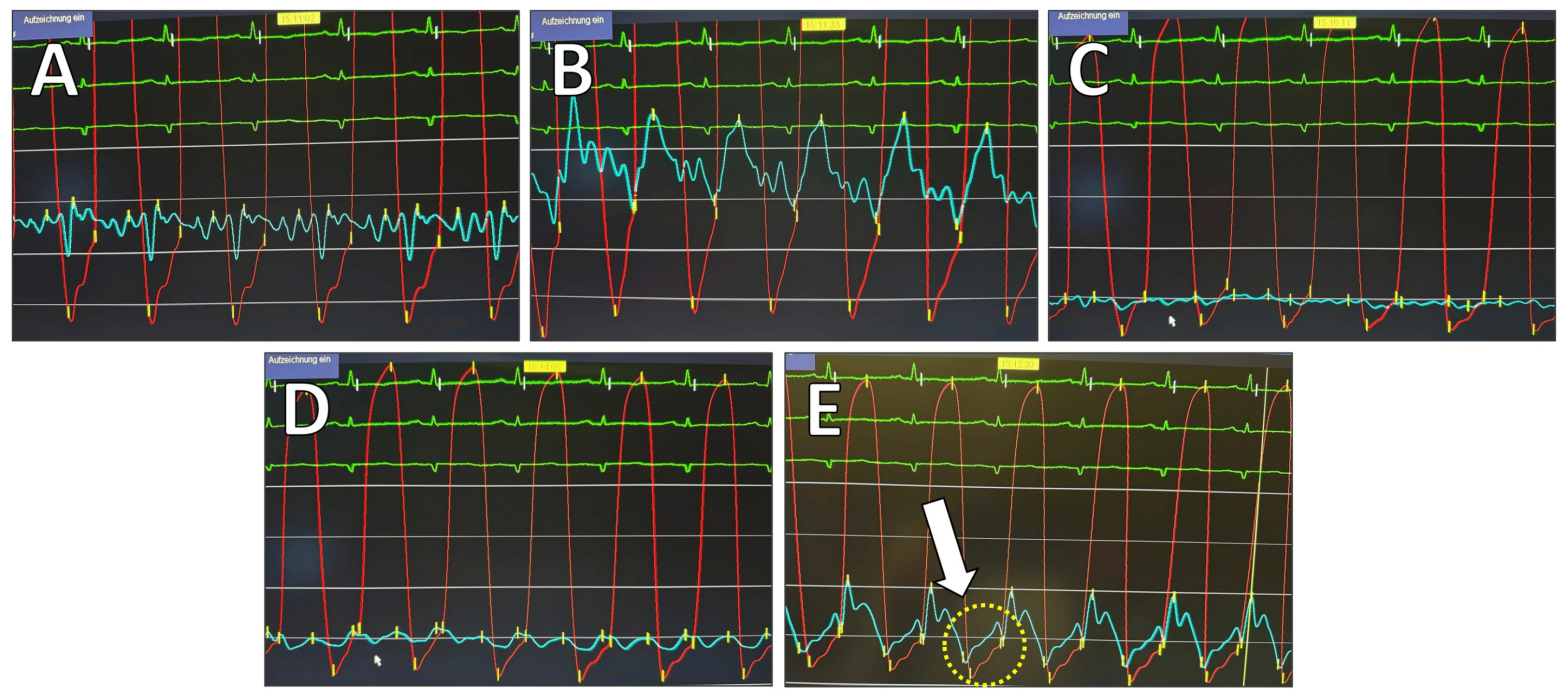
Hemodynamic pressure tracings illustrating the classic findings of constrictive pericarditis. (A) Simultaneous left ventricular (LV) and pulmonary capillary wedge pressure (PCWP) recordings. (B) LV and pulmonary artery (PA) pressure tracings. (C) LV and superior vena cava (SVC) pressure relationship. (D) LV and right atrial (RA) pressure tracings, demonstrating elevated RA pressure. (E) LV and right ventricular (RV) pressure curves, showing the characteristic dip-plateau (square root) sign (arrow).

Transthoracic echocardiography (TTE) revealed a persistently thickened pericardium with organized pericardial effusion measuring between 5 and 10 mm, right ventricular dysfunction, and a restrictive filling pattern (Figure 1). To further assess the structural changes of the pericardium, a contrast-enhanced CT scan of the thorax was performed, demonstrating a 9 mm circumferential pericardial thickening with signs of calcification and a small pericardial effusion. Additionally, findings were consistent with bilateral pleural effusions and right heart strain, indicated by contrast reflux into the inferior vena cava and hepatic veins. No evidence of pulmonary embolism was found, but a mildly enlarged right ventricle suggested ongoing pressure overload (Figure 2). To further evaluate the hemodynamic impact, right and left heart catheterization was performed, demonstrating significantly elevated and equalized diastolic pressures across all cardiac chambers (pulmonary capillary wedge pressure (PCWP) = pulmonary artery diastolic pressure (PAdiast) = left ventricular end-diastolic pressure (LVEDP) = right ventricular end-diastolic pressure (RVEDP) = right atrial mean pressure (RAmitt)) (Table 1). The right ventricular pressure curve exhibited a characteristic dip-plateau phenomenon, further supporting the diagnosis of CP (Figure 3). Given the patient’s progressive heart failure symptoms, imaging findings, and hemodynamic compromise, he was referred for surgical intervention.

**Table 1:** Invasive hemodynamic measurements in constrictive pericarditis (CP). This table presents the invasive pressure measurements obtained during right and left heart catheterization, demonstrating the classic hemodynamic profile of constrictive pericarditis. The pulmonary capillary wedge pressure (PCW) shows elevated mean pressures, consistent with impaired ventricular compliance. The pulmonary artery (PA) pressures indicate mild pulmonary hypertension. The right ventricular (RV) pressure tracing reveals a characteristic dip-plateau phenomenon, with an elevated end-diastolic pressure (RVEDP), nearly equal to the left ventricular end-diastolic pressure (LVEDP). The right atrial pressures (RAPs) are significantly elevated, demonstrating restricted right heart filling. The left ventricular (LV) pressure profile shows a preserved systolic function but markedly increased LVEDP, indicative of diastolic dysfunction due to pericardial constriction. The aortic (AO) pressures remain within normal systemic ranges, highlighting that the primary pathophysiology is related to ventricular filling abnormalities rather than outflow obstruction. The findings are consistent with the hemodynamic hallmark of CP, in which all diastolic pressures are nearly equalized (PCW ≈ RVEDP ≈ LVEDP ≈ RAP), confirming severe ventricular constraint due to the thickened, fibrotic pericardium.

Measured Cavity	Measured Values (mmHg)	Normal Reference Range (mmHg)
PCW (a/v/mean)	28/26/25	a: 6-12, v: 6-12, mean: 6-12
PA (systolic/diastolic/mean)	45/26/34	S: 15-30, D: 4-12, M: 9-19
RV (systolic/diastolic/EDP)	48/15/24	S: 15-30, D: 3-8, EDP: <8
RA (a/v/mean)	27/25/23	a: 2-8, v: 2-8, mean: 2-6
LV (systolic/diastolic/EDP)	148/7/24	S: 100-140, D: 3-12, EDP: 5-12
AO (systolic/diastolic/mean)	136/11/61	S: 100-140, D: 60-90, Mean: ~100

**Figure 4:**
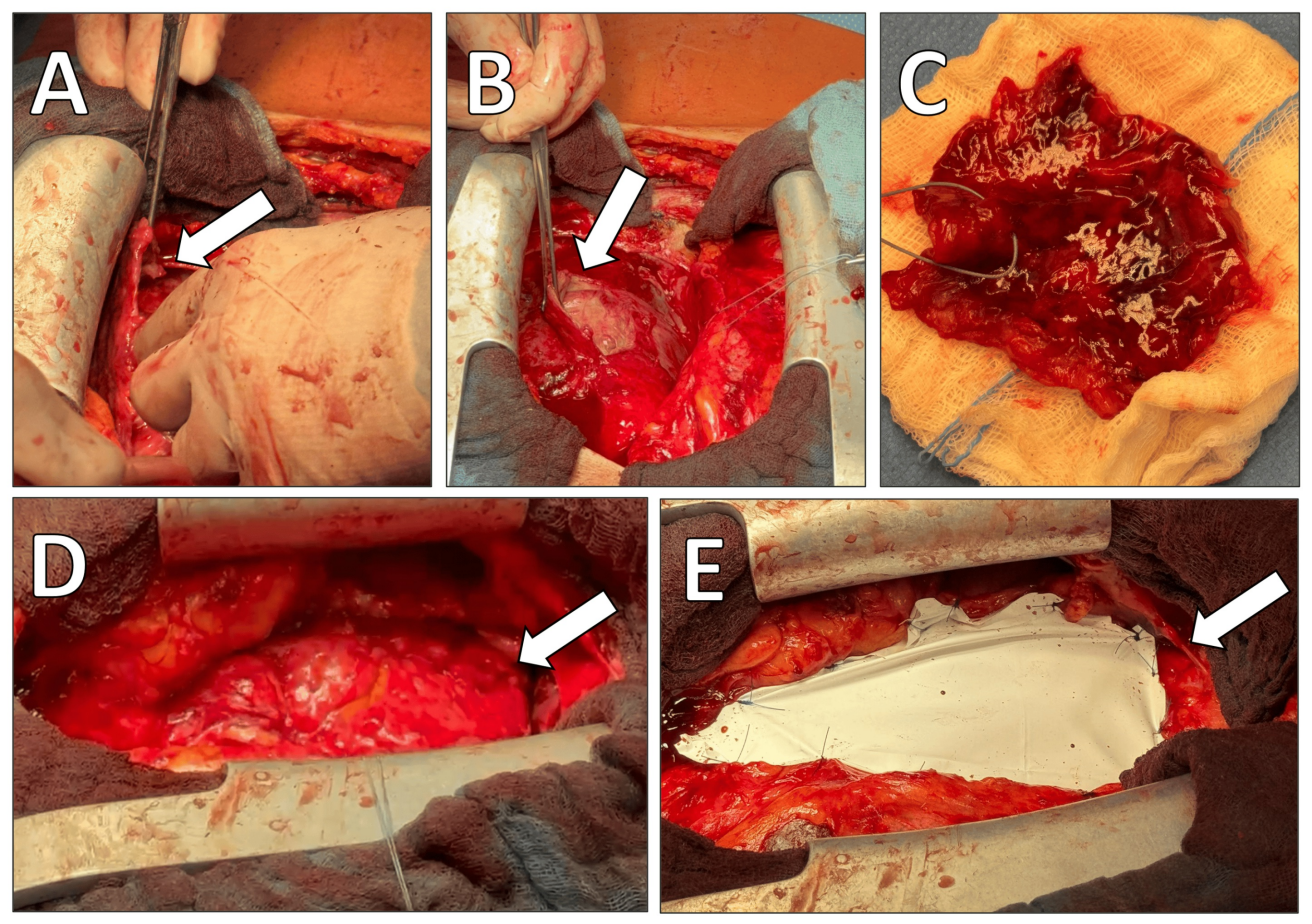
Intraoperative images depicting key surgical steps in pericardiectomy. (A) Identification of the thickened pericardium (arrow). (B) Careful dissection and mobilization of the pericardium from the underlying myocardium (arrow). (C) Resected pericardial tissue specimen. (D) Restoration of normal myocardial contractility following pericardial release (arrow). (E) Placement of a Gore-Tex membrane (W. L. Gore & Associates, Newark, Delaware, USA) to minimize postoperative adhesions (arrow).

The patient was taken to the operating room, where he underwent a subtotal pericardiectomy with pericardial reconstruction using a Gore-Preclude membrane (W. L. Gore & Associates, Newark, Delaware, USA) under general anesthesia. Intraoperative transesophageal echocardiography (TEE) confirmed impaired left ventricular function without significant valvular disease. A midline sternotomy was performed, exposing the pericardium, which appeared markedly thickened and fibrotic. A pericardial incision was carefully made over the ascending aorta, revealing extensive adhesions between the pericardium and the underlying myocardium, consistent with CP. Given the significant pericardial involvement, a meticulous pleural adhesiolysis was undertaken, allowing for complete mobilization of the heart. The thickened pericardium was carefully resected, ensuring adequate circumferential decompression of the ventricles while avoiding injury to the coronary arteries and myocardial tissue. To prevent postoperative adhesion formation, a 0.1 mm Gore-Preclude membrane was cut and loosely secured to the mediastinum using 3-0 Prolene sutures (Ethicon, Inc., New Brunswick, New Jersey, USA) (Figure 4).

In the immediate postoperative period, the patient remained hemodynamically stable with controlled inotropic support. He was successfully extubated on postoperative day 3 and gradually weaned off vasopressors. A TTE assessment at this stage showed improved left ventricular filling dynamics, resolution of the restrictive pattern, and no significant pericardial effusion. By the end of the first postoperative week, the patient demonstrated marked clinical improvement with reduced jugular venous distension, regression of ascites, and resolution of peripheral edema. The pleural and pericardial drains were removed on postoperative day 7 after a significant decrease in drainage output. The patient progressively regained mobility and achieved full ambulation by postoperative day 10.

The final echocardiographic assessment before discharge confirmed the absence of pericardial effusion, normalization of right ventricular function, and complete resolution of ventricular interdependence, indicating effective pericardial decompression. The patient was discharged on postoperative day 11 in stable condition with well-controlled hemodynamics and no signs of heart failure. A follow-up plan was established, including antiplatelet therapy, beta-blockers, and consideration of colchicine (0.5 mg twice a day) therapy to prevent recurrent inflammation.

## Discussion

CP is a progressive condition characterized by fibrotic thickening and adherence of the pericardium to the myocardium, leading to impaired diastolic filling and reduced cardiac output. This results in chronic venous congestion, progressive heart failure symptoms, and significant hemodynamic compromise [1-3]. While CP can develop from various etiologies, infectious pericarditis, particularly purulent pericarditis, is among the most severe and rapidly progressing causes [5]. The present case illustrates the complex interplay between infectious pericarditis, chronic inflammation, and fibrotic remodeling, ultimately leading to the development of clinically significant CP requiring surgical intervention.

Pathophysiology and hemodynamic features of CP

The hallmark of CP is diastolic dysfunction due to a noncompliant pericardium. The fibrotic pericardium restricts ventricular expansion, leading to the equalization of diastolic pressures across all cardiac chambers. These hemodynamic findings reflect early rapid diastolic filling, followed by an abrupt cessation due to the pericardial constraint, a key feature distinguishing CP from restrictive cardiomyopathy. Hemodynamic assessment thus plays a crucial role in diagnosing CP. In the present case, right and left ventricular pressure measurements demonstrated a significant increase in diastolic pressures, with the characteristic dip-plateau (square root) sign, indicating restrictive diastolic filling. The right ventricular end-diastolic pressure (RVEDP) was approximately one-third of the systolic RV pressure, which supports constrictive physiology. The left ventricular end-diastolic pressure (LVEDP) was nearly equal to the pulmonary capillary wedge pressure (PCWP), pulmonary artery diastolic pressure (PAD), and mean right atrial pressure (RAm), reflecting the classic diastolic pressure equalization seen in CP. These findings confirm the hallmark hemodynamic profile of CP, in which ventricular filling is abruptly halted due to pericardial constraint, leading to diastolic pressure equalization across all cardiac chambers. Additionally, the right atrial pressure curve exhibited an "M" or "W" configuration, reflecting impaired right atrial compliance, and Kussmaul’s sign (paradoxical rise in jugular venous pressure during inspiration, indicating impaired right ventricular filling), further supporting the diagnosis. The presence of mildly elevated pulmonary artery pressures (<45 mmHg) suggests secondary pulmonary involvement, often observed in advanced CP. The dip-plateau phenomenon, also known as the "square root sign," is a characteristic hemodynamic finding in CP, reflecting the abrupt cessation of ventricular filling due to pericardial constraint. It is observed in ventricular pressure tracings as an initial rapid early diastolic decline (the "dip") followed by a plateau phase, indicating a sudden limitation in diastolic expansion (Table 2). These invasive pressure measurements, in combination with echocardiographic and imaging findings, played a pivotal role in differentiating CP from restrictive cardiomyopathy and guiding the decision for surgical intervention [6-9].

**Table 2:** Typical hemodynamic findings in pressure curves of constrictive pericarditis. RVEDP: right ventricular end diastolic pressure; LVEDP: left ventricular end diastolic pressure; PCWPm: mean pulmonary capillary wedge pressure; PAD: diastolic pulmonary artery pressure; RAm: mean right atrial pressure; RV: right ventricular; LV: left ventricular; PA: pulmonary artery

Parameter	Finding
Ventricle pressure	Dip-plateau phenomenon in RV and LV, early diastolic pressure increased to 5-10 mmHg
RV pressure	RVEDP 1/3 of the systolic RV pressure
Filling pressures	Elevated: LVEDP=PCWPm=PAD=RVEDP=RAm
PA pressure	Systolic PA pressure <45 mmHg
RA pressure	"M" or "W" configuration, Kussmaul's phenomenon
LV angiography	Normal ejection fraction (EF), reduced stroke volume, pericardial calcifications in 40% of the cases
Coronary angiography	Normal findings

Another critical feature seen in this patient was right ventricular dysfunction with elevated right atrial pressures, resulting in jugular venous distension, hepatomegaly, ascites, and peripheral edema. The congestive symptoms, rather than a low-output state, often dominate the clinical presentation of CP, making it essential to differentiate from other causes of heart failure [10]. The absence of significant pulmonary congestion and the presence of right-sided overload further supported the diagnosis. Additionally, septal bounce and ventricular interdependence on echocardiography provided crucial non-invasive evidence of CP, reinforcing the need for surgical intervention.

The role of multimodal imaging in CP diagnosis

While echocardiography remains the initial imaging modality, CT and cardiac MRI provide valuable complementary information in CP assessment [2,11]. In our patient, CT imaging revealed circumferential pericardial thickening (9 mm), pericardial calcifications, and a small but organized pericardial effusion. These findings, along with the presence of pleural effusions and signs of right heart strain, strongly suggested advanced pericardial constriction. CT also allowed for the exclusion of pulmonary embolism as a potential contributor to the patient’s symptoms. The use of multimodal imaging is particularly critical in distinguishing CP from restrictive cardiomyopathy, a differential diagnosis with overlapping clinical and hemodynamic features but distinct treatment strategies [10].

Surgical management: pericardiectomy as the definitive therapy

Pericardiectomy is the only definitive treatment for CP, aiming to remove the fibrotic pericardium and restore normal cardiac filling dynamics. The timing of surgery is crucial, as prolonged pericardial constriction can lead to irreversible myocardial fibrosis, persistent diastolic dysfunction, and long-term right ventricular failure, reducing the likelihood of complete hemodynamic recovery [12]. Our patient exhibited significant clinical symptoms and clear hemodynamic evidence of severe CP, making him a strong surgical candidate.

The surgical approach involved a midline sternotomy, extensive pericardial adhesiolysis, and subtotal pericardiectomy, ensuring maximal cardiac decompression while preserving myocardial integrity [4]. One of the intraoperative challenges in CP surgery is the presence of dense adhesions, which increase the risk of coronary injury and ventricular perforation. In this case, a meticulous stepwise approach was employed, carefully mobilizing the heart and resecting the fibrotic pericardium to release the ventricles circumferentially. A Gore-Preclude pericardial membrane was placed to minimize postoperative adhesion formation and facilitate future re-operations, if needed.

Postoperatively, the patient demonstrated rapid hemodynamic improvement, with echocardiographic findings confirming the resolution of the restrictive filling pattern and normalization of right ventricular function. He was successfully weaned off inotropic support and achieved full ambulation by postoperative day 10, highlighting the effectiveness of early surgical intervention in restoring normal cardiovascular function.

Prognostic considerations and postoperative outcomes

The prognosis of CP following pericardiectomy depends on several factors, including the extent of pericardial involvement, duration of constriction, and preoperative myocardial function [9]. Patients with long-standing CP and severely impaired right ventricular function may experience persistent heart failure symptoms despite surgical intervention, emphasizing the importance of early diagnosis and referral for surgery. In this patient, the absence of significant myocardial dysfunction preoperatively contributed to a favorable postoperative course.

However, pericardiectomy carries a non-negligible perioperative risk, with mortality rates ranging from 4% to 16% depending on the severity of CP and underlying comorbidities [1,12-14]. Common postoperative complications include low cardiac output syndrome, arrhythmias, and recurrent effusions, which require close monitoring in the immediate postoperative period [1,15]. Our patient did not develop any major complications, and his final echocardiographic assessment demonstrated normalization of biventricular function, complete relief of pericardial constriction, and no residual pericardial effusion.

Long-term follow-up is essential, as some patients may experience persistent diastolic dysfunction due to pre-existing myocardial fibrosis [16]. In this case, colchicine therapy was considered as an adjunctive measure to reduce the risk of recurrent pericardial inflammation. Continued clinical and echocardiographic monitoring will be necessary to ensure sustained improvement.

## Conclusions

This case highlights the complex interplay between infectious pericarditis, pericardial fibrosis, and hemodynamic deterioration, ultimately leading to the development of symptomatic CP requiring pericardiectomy. The diagnosis of CP relies on a combination of clinical findings, multimodal imaging, and invasive hemodynamic assessment, with cardiac catheterization playing a crucial role in confirming the pathophysiologic hallmark of equalized diastolic pressures and the dip-plateau phenomenon. Pericardiectomy remains the gold standard treatment, offering significant symptomatic relief and improved long-term outcomes when performed in a timely manner. The successful postoperative recovery of our patient demonstrates the importance of early intervention to prevent irreversible myocardial dysfunction and optimize hemodynamic recovery. Given the risk of recurrence, residual diastolic dysfunction, or late complications, close long-term follow-up with clinical and echocardiographic assessment is essential.
